# The impact of drug resistance on *Mycobacterium tuberculosis* physiology: what can we learn from rifampicin?

**DOI:** 10.1038/emi.2014.17

**Published:** 2014-03-12

**Authors:** Anastasia Koch, Valerie Mizrahi, Digby F Warner

**Affiliations:** Medical Research Council/National Health Laboratory Service/University of Cape Town Molecular Mycobacteriology Research Unit, Department of Science and Technology/National Research Foundation Centre of Excellence for Biomedical Tuberculosis Research, Institute of Infectious Disease and Molecular Medicine and Department of Clinical Laboratory Sciences, University of Cape Town, Cape Town 7701, South Africa

**Keywords:** epistasis, fitness cost, RNA polymerase, *rpoB*, TB

## Abstract

The emergence of drug-resistant pathogens poses a major threat to public health. Although influenced by multiple factors, high-level resistance is often associated with mutations in target-encoding or related genes. The fitness cost of these mutations is, in turn, a key determinant of the spread of drug-resistant strains. Rifampicin (RIF) is a frontline anti-tuberculosis agent that targets the *rpoB*-encoded β subunit of the DNA-dependent RNA polymerase (RNAP). In *Mycobacterium tuberculosis* (*Mtb*), RIF resistance (RIF^R^) maps to mutations in *rpoB* that are likely to impact RNAP function and, therefore, the ability of the organism to cause disease. However, while numerous studies have assessed the impact of RIF^R^ on key *Mtb* fitness indicators *in vitro*, the consequences of *rpoB* mutations for pathogenesis remain poorly understood. Here, we examine evidence from diverse bacterial systems indicating very specific effects of *rpoB* polymorphisms on cellular physiology, and consider these observations in the context of *Mtb*. In addition, we discuss the implications of these findings for the propagation of clinically relevant RIF^R^ mutations. While our focus is on RIF, we also highlight results which suggest that drug-independent effects might apply to a broad range of resistance-associated mutations, especially in an obligate pathogen increasingly linked with multidrug resistance.

## INTRODUCTION

The discovery of antibiotics in the 1940s revolutionized the treatment of infectious diseases and, at the same time, suggested the possibility of eradicating bacterial pathogens as a major cause of morbidity and mortality.^1^ The intervening 70 years have, however, seen the emergence of organisms which are resistant to almost every antibiotic that has been introduced into mainstream use.^1,2^ Tuberculosis (TB) is no exception: in 2012, there were an estimated 450 000 cases of multidrug-resistant (MDR) TB, which is defined as disease caused by strains of *Mycobacterium tuberculosis* (*Mtb*) that are resistant to the frontline anti-TB drugs, isoniazid (INH) and rifampicin (RIF). Almost 10% of these MDR cases were classified as extensively drug-resistant (XDR) TB,^3^ in which there is additional resistance to any of the fluoroquinolone (FQ) antibiotics and at least one of the second-line injectable aminoglycosides—amikacin, capreomycin or kanamycin. Although accounting for a small number of cases relative to drug-susceptible disease, drug-resistant TB imposes a disproportionate burden on public health systems—especially in endemic regions^4^—and, for this reason, MDR and XDR *Mtb* strains are considered emerging pathogens in their own right.^5^ However, while there has been intensive analysis of the risk factors—primarily social and programmatic—driving the emergence of resistance,^6,7,8^ the global burden of drug-resistant TB continues to increase. Effective TB control will require a deeper understanding of the impact of drug resistance on the host–pathogen interaction and of the biological factors underlying the relative success of drug-resistant strains.^9^

**Figure 1:**
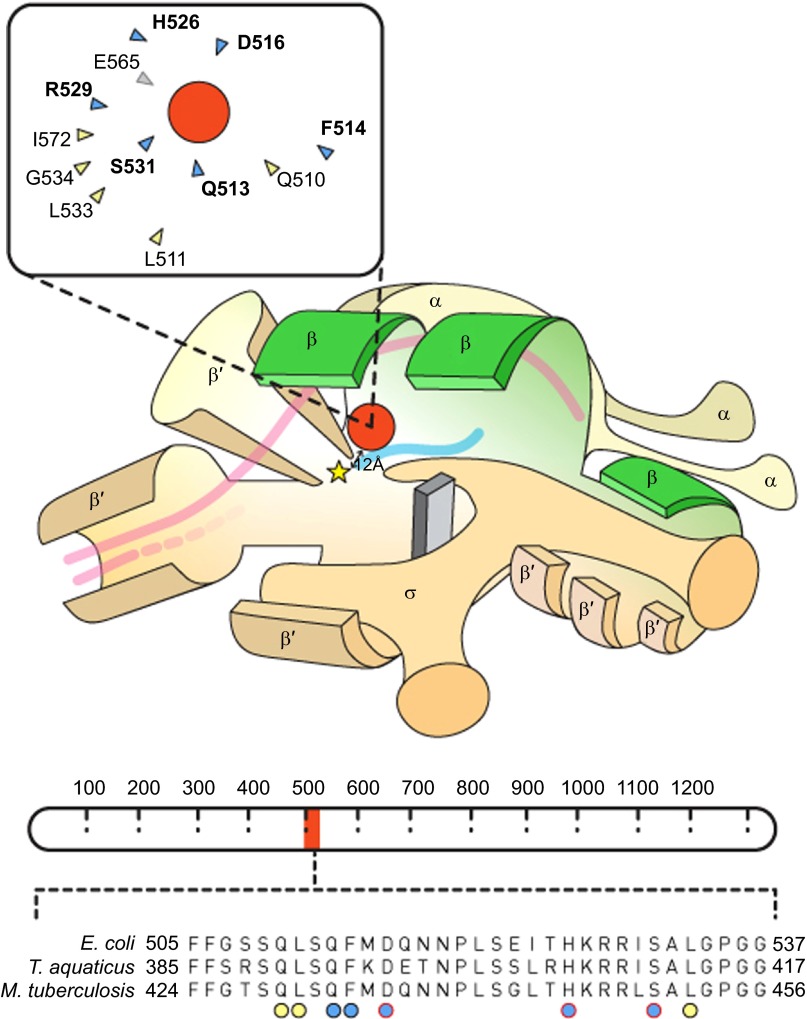
Schematic representation of RNAP structural elements including the RIF resistance determining region (RRDR). The cartoon showing the RNAP holoenzyme is adapted from Borukhuv and Nudler.^17^ Structural annotations have been simplified, and the promoter sequence has been excluded. The *rpoB*-encoded β subunit is highlighted in green. A yellow star represents the RNAP active site and a red circle denotes the RIF molecule which approaches within 12 Å of the active site,^18^ inhibiting transcription. Double-stranded DNA is represented by pink lines and, once unwound, only template DNA is shown, with the growing RNA chain colored in blue. The inset shows a simplified depiction of the RIF binding pocket.^18^ Amino acids that form hydrogen bonds with RIF are highlighted in blue and those that form van der Waals interactions are colored yellow; amino-acid numbering corresponds to that used for *E. coli*. Mutations identified in 11 of the 12 residues that surround the RIF binding pocket have been associated with RIF resistance, albeit at different frequencies^18^ (the sole amino acid, E565, which has not been associated with RIF^R^ mutations is colored in grey). A schematic representation of the *rpoB* gene which encodes the β subunit of RNA polymerase is shown below the RNAP cartoon (adapted from Campbell *et al.*^18^). Amino-acid numbering is shown as dashed demarcations. The RRDR is highlighted in blue and the amino-acid sequence of the RRDR is magnified below. The alignment contains the amino-acid sequences of *E. coli*, *T. aquaticus* and *M. tuberculosis*. Amino acids that interact directly with RIF are indicated by circles and the colors correspond to the inset diagram. Circles highlighted in red indicate residues that are most frequently observed in RIF^R^ isolates.^18^

Comparative genomic analyses have established that high-level drug resistance in *Mtb* arises almost exclusively through chromosomal mutations in genes required for antibiotic action,^10,11,12,13,14,15^ that is, genes encoding the protein targets of the applied drugs, or the enzymes required for prodrug activation. Since antibiotics target essential cellular functions, it might be expected that resistance mutations in target-encoding genes will impact pathogenesis—a concept loosely captured in the term ‘fitness cost'.^16^ In turn, this raises fundamental questions regarding the ability of *Mtb* to harbour multiple drug resistance mutations while retaining the ability to infect, persist, and cause disease in its obligate human host. We are interested in RIF resistance (RIF^R^), which results primarily from single-nucleotide substitution mutations in a small region of *rpoB*, the gene encoding the β-subunit of the DNA-dependent RNA polymerase (RNAP) (Figure 1). Given the essentiality of RNAP for transcription, it appears likely that mutations in *rpoB* will have multiple effects on *Mtb* physiology in addition to RIF^R^. In this review, we summarize insights obtained from other bacterial systems into the structural and physiological consequences of *rpoB* mutations, and consider these observations in the context of the available evidence from *Mtb*. In addition, we assess the potential impact of RIF^R^ on *Mtb* physiology and pathogenesis and discuss the possible consequences for the continued emergence of drug resistance in a pathogen that is uniquely adapted to human infection.^19^

## WHY IS RIF IMPORTANT FOR TB?

Together with INH, RIF is a major frontline anti-TB agent and has been included in standard chemotherapy since the 1980s.^20^ RIF is also used for the treatment of asymptomatic *Neisseria meningitides* carriers and, like other rifamycins, has been prescribed for *Streptococcus pneumoniae*, *Legionella pneumophila* and opportunistic *Rhodococcus equi* infections.^21^ Moreover, the emergence of methicillin resistance and, more recently, vancomycin resistance (VAN^R^) has resulted in the increasing application of RIF for *Staphylococcus aureus* infections.^21^ Unlike most current antibiotics which require active growth and metabolism to exert their anti-bacterial effects,^22^ RIF is included in a select category of agents (other examples include moxifloxacin and bedaquiline), which retain activity against slow-growing, and even non-replicating, *Mtb* bacilli.^23,24,25^ This property is especially important for TB, where low metabolic activity and/or non-replication are considered key factors in persistent *Mtb* infection.^26,27,28^ In fact, the role of RIF in sterilizing slowly metabolizing bacillary populations^20^ is a major factor in the continued reliance of public health programmes on RIF as a frontline anti-TB drug, despite the emergence and spread of RIF^R ^*Mtb* strains.^29^

### How does RIF kill bacteria?

Most bacteria possess a single DNA-dependent RNAP enzyme comprising a multisubunit αββ′ω core that forms a ‘crab claw-like' structure.^17,30,31^ The β and β′-subunits constitute the main components of each ‘pincer' of the claw, forming a groove that accommodates the template DNA and provides a catalytic site for phosphodiester bond formation, a secondary channel for incoming nucleotides, and a separate exit for the growing RNA transcript.^17,30,31^ In a tight complementary fit, RIF binds to the *rpoB*-encoded β-subunit, thereby inhibiting transcription (Figure 1). Structural analysis of the *Thermus aquaticus* RNAP has shown that the RIF binding site is located within the DNA/RNA channel, but not at the active site.^18^ Moreover, RIF-bound RNAP retains the ability to catalyse formation of the first phosphodiester bond in a nascent RNA transcript, suggesting that RIF does not inhibit catalysis. Instead, it seems that the drug obstructs the path of a growing RNA chain of two to three nucleotides in length: once transcriptional elongation is in full progress, RNAP is no longer vulnerable to RIF-mediated inhibition. For this reason, RIF activity is restricted to a very specific stage of transcription.^18^ However, the precise mechanism by which RIF-mediated transcriptional interference leads to cell death is not well understood.

### Mutations in *rpoB* confer RIF^R^

Early studies in *Escherichia coli* mapped RIF^R^ mutations to three distinct clusters (I, II and III) within the *rpoB* gene.^18^ It has subsequently been shown that, across all bacterial species, the majority of RIF^R^ mutations occur in an 81 bp region of cluster I—the so-called RIF resistance determining region (RRDR)^32,33,34^—though some mutations have been identified in other regions of *rpoB* as well.^35,36^ As the RRDR was originally defined in *E. coli*, it is standard practice to use that organism's numbering system even when describing specific features of RpoB from other bacteria;^37^ that tradition is observed for the remainder of this article.

Of the 12 amino acids that surround the RIF binding pocket, 11 have been associated with RIF^R^ mutations (Figure 1).^18^ From analyses of clinical *Mtb* isolates from various geographical regions, it is evident that specific RIF^R^ mutations are identified more frequently than others.^12^ However, across all bacterial species, RIF^R^ is generally associated with mutations in the binding pocket that substitute an amino acid with a compact side chain with one that is larger (e.g., Ser→Leu), thereby preventing access of the relatively inflexible RIF molecule to RNAP.^18^ In *E. coli* and *Bacillus subtilis*, *rpoB* mutations have been shown to impact promoter binding^38,39^ and transcriptional elongation and termination,^40,41,42,43,44,45,46^ as well as elements of transcription-coupled repair.^47,48,49^ So profound are these effects that *rpoB* mutations have been exploited to examine mechanisms of transcription since well before there was an interest in the clinical implications of RIF^R^.^33^

There have also been reports of RIF-dependent *E. coli* strains.^50^ Similarly, enhanced growth of some *Mtb* mutants has been observed *in vitro* in the presence of RIF^51^ and was recently described in a clinical *Mtb* strain obtained from a patient whose TB symptoms deteriorated while undergoing treatment with a regimen containing rifamycins.^52^ The infecting *Mtb* isolate—carrying the common *rpoB* S531L mutation together with a less frequently observed F584S polymorphism—exhibited improved growth in RIF-containing *versus* antibiotic-free growth medium. There is some analogy between this observation and the identification of streptomycin (STR)-dependent clinical isolates such as *Mtb* 18b, a strain whose inability to grow in the absence of STR has recently been exploited as a useful model for TB drug discovery.^25^ However, whereas the STR dependence of 18b is thought to result from an insertion mutation in the 16S rRNA sequence that is stabilized by the STR molecule,^53^ the mechanism(s) underling RIF-dependent growth remains unclear. The presence of an F584S mutation in addition to S531L in the RIF-dependent mutant implies that secondary mutations might contribute to the phenotype of strains that grow better in the presence of RIF; however, this requires formal demonstration. Moreover, it is not known just how often the phenomenon of improved growth in the presence of RIF occurs clinically, notwithstanding its potential implications for TB diagnosis and treatment.

In *Mtb*, c→t transitions underlie two of the most commonly observed *rpoB* mutations: S531L (tcg→ttg) and H526Y (cac→tac).^12,54^ Spontaneous deamination of cytosine to uracil occurs readily in all living cells^55^ and the high G+C-content of *Mtb* genomic DNA probably renders the organism especially susceptible to these events.^56^ Moreover, it is possible that this effect is exacerbated by host immune defences, which include the production of reactive oxygen and nitrogen intermediates^57^ and, for *rpoB* in particular, is further amplified by the selective advantage of the S531L mutation.^58^ There is, however, important recent evidence that, for *Mtb*, the number of potential RIF^R^-conferring mutations in *rpoB*—the effective ‘target size' for resistance—can differ according to strain lineage, at least *in vitro*.^59^ This effect is most clearly observed at lower RIF concentrations and, in combination with separate studies identifying putative ‘low-level' resistance and/or compensatory mutations in clinical isolates,^10,11^ suggests that further work is necessary to determine the contribution of ‘rare' *rpoB* alleles—and other infrequent resistance-associated mutations—to the emergence of drug resistance. For example, mutations have been identified outside the RRDR in clinical RIF^R^ *Mtb* strains.^60^ However, given the focus of genotypic assays on the RRDR for detecting RIF^R^,^61^ it is inevitable that any non-RRDR mutations will be underrepresented in molecular epidemiological surveys of resistance.

The conditions favouring the emergence of specific mutations are also poorly understood: for example, it has been shown that the diversity of *rpoB* mutations in *E. coli* differs depending on the rate at which susceptible organisms are exposed to increasing RIF concentrations.^62^ Similarly, adjusting the pH of a chemostat culture of *Mtb* results in a different spectrum of *rpoB* mutations from that which is commonly observed during growth under standard conditions *in vitro*,^63^ perhaps suggesting the role of a specific selective pressure(s) in ensuring fixation (and even propagation) of the lowest cost mutations.

## THE FITNESS COST OF RIF^R^ MUTATIONS

In general, resistance mutations in essential genes have a negative impact on key physiological functions. That is, drug resistance incurs a fitness cost: in the absence of drug pressure, strains without resistance-associated mutations are generally better able to complete the bacterial life cycle in a variety of growth conditions.^2,64,65,66,67^ The cost of resistance has been identified as a critical determinant of the spread of drug-resistant *Mtb* strains.^68^ Given the clinical importance of RIF, it is not surprising that the fitness cost of *rpoB* mutations has been the subject of intense investigation, both in *Mtb* and other organisms.^65,69,70^ Overall, these studies have reported that the fitness of specific *rpoB* mutants relative to the corresponding drug-susceptible parental strain depends primarily on the strain background and the nature of the assay employed to assess fitness.^64,66,67,71,72,73^ However, in most cases, strain ‘fitness' is conflated with growth rate under a limited set of *in vitro* conditions, probably because of the relative ease of performing—and interpreting—bacterial growth assays. In their natural environments, most bacteria seldom encounter conditions which allow for uninterrupted exponential growth.^74^ Therefore, alternative fitness models might be required to recapitulate the selective pressures that impact competitive fitness, such as adaptation to stationary-phase *in vitro*^75^ (discussed below) or virulence in experimental models of infection.^76,77^ This issue is explored further below (see ‘*IN VITRO* FITNESS AS A SURROGATE FOR PATHOGEN SUCCESS?' section).

### Strain fitness *versus* epidemiological success

It is likely, too, that for obligate pathogens such as *Mtb*, growth rate is just one of many factors that might contribute to the ‘success' of the organism. That is, multiple fitness attributes will influence the short-term competitiveness of specific *Mtb* mutants (or lineages) and, in turn, their longer-term evolution within different hosts and host populations. Therefore, for the purposes of this review, the term ‘fitness' might be defined as the inherent capacity of an organism to drive its lifecycle, the composite phenotype that represents the sum of all genetic and physiological features and abilities. In contrast, ‘success' might be considered the outcome of multiple contributing ‘fitness' attributes, and should be measured by the longevity of a pathogen within a specific environment or host population. For an obligate pathogen, the fitness attributes which contribute to the success of specific lineages (drug-resistant or not) might be usefully considered in terms of discrete steps of the infection lifecycle: that is, the ability to establish an infection, and to replicate and persist within a host, as well as the capacity for transmission.

In turn, this suggests that any resistance cost should be considered in the context of longer-term pathogen success, which is itself a function of multiple biological processes that might not directly influence growth rate, but could impact other core physiological functions. The recent observation that multiple small-effect mutations might contribute to the stepwise emergence of drug resistance or to reducing the cost of acquired resistance,^10,11,78^ appears to reinforce this notion; such polymorphisms could be critical to the ability of the organism to survive very specific selective pressures that do not correlate with relative growth rate *in vitro*.

### The impact of compensatory mutations

Compensatory mutations in target or related genes can reduce the fitness costs of drug resistance.^9,67,79^ In a landmark study, Gagneux *et al.*^67^ measured the growth rates of RIF^R^ *Mtb* mutants relative to drug-susceptible parental strains. Their experiments utilized spontaneous RIF^R^ mutants selected *in vitro*, as well as clinical isolates obtained from TB patients who had developed drug resistance. The strains covered two of the seven main *Mtb* lineages^80^ and the results revealed that competitive fitness was dependent not only on the nature of the *rpoB* mutation (e.g. S531L *versus* H526Y), but also strain genotype: fitness differed considerably between lineages even where the *rpoB* mutation was identical.^67^ As suggested by the authors, these data implied that the presence (or absence) of compensatory mutations might contribute to the inferred fitness cost.^67^ To address this possibility, the same group recently examined a large database of RIF^S^ and RIF^R^ clinical isolates for compensatory mutations.^79^ Their analyses revealed that a significant proportion of RIF^R^ strains carried mutations in *rpoA* or *rpoC*—encoding the RNAP α and β′ subunits, respectively—whereas the same mutations were not found in susceptible isolates. This observation, together with the location of the polymorphisms in subunits that interact closely with *rpoB* in the RNAP holoenzyme (Figure 1), identified the observed mutations as compensatory.^79^ Consistent with this notion, an elevated frequency of *rpoC* mutations was recently described in closely related strains from South Africa, suggesting a potential association between the propagation of RIF^R^ strains and the presence of *rpoC* mutations.^81^ Similar panels of *rpoC* and *rpoB* mutations have subsequently been identified among RIF^R^ clinical *Mtb* isolates in multiple separate studies, reinforcing the inferred role of multiple RNAP mutations in conferring RIF^R^ while maintaining strain fitness.^10,11,13^

The selective pressure for the acquisition of compensatory mutations to restore competitive fitness is further supported by similar observations in *Salmonella enterica*, where the acquisition of *rpoA* and *rpoC* mutations has been shown to improve the growth rate of slow-growing *rpoB* mutants during serial passage in media containing RIF.^70,82^ In that case, though, the authors confirmed the necessity and sufficiency of the identified mutations for reversal of the growth defect by introducing the same mutations into the chromosome of the original *rpoB* mutant, thereby providing genetic validation of the inferred compensatory effect. Interestingly, the *rpoA* and *rpoC* mutations did not alter the susceptibility of the *rpoB* mutant to RIF, yet the same mutations were associated with small but significant decreases in RIF susceptibility when introduced into the fully susceptible wild-type strain. The combination of *Mtb* and *S. enterica* studies strongly suggests that *rpoA* and *rpoC* mutations are compensatory;^82^ however, key questions remain. For example, given that the *rpoB* mutation identified in the *S. enterica* study (R529C) is not frequently detected in clinical *Mtb* isolates,^12^ what is the role of compensatory mutations in strains carrying more frequently observed *rpoB* alleles? Also, what are the structural and/or molecular mechanisms underlying the inferred compensatory effects? Comparing the impact of compensatory mutations on diverse physiological and pathogenic features in isogenic strains will be critical to understand the selective advantage conferred by the identified RNAP polymorphisms.

### A role for epistasis?

Independent resistance mutations can interact to influence the fitness of drug-resistant bacteria. This concept is captured in the term ‘epistasis', which might be defined broadly as an interaction between genes,^83^ but is often used to refer to the masking of phenotypic effects of one allele by another allele. To avoid confusion, the term epistasis is used throughout this review to denote the dependence of the phenotypic expression of one allele on another, distinct allele.^84^ For example, strong positive epistasis has been described for genes associated with resistance to STR, RIF and nalidaxic acid in *E. coli*.^84^ Similarly, a very recent *in vitro* study of engineered FQ-resistant (FQ^R^) *Salmonella* mutants has demonstrated synergistic epistasis between specific *gyrA* and *parC* alleles.^85^ Critically, these results provide direct evidence of the potential for drug resistance-associated mutations to confer a strong fitness advantage, even in the absence of antibiotic selection.

The interaction of drug resistance determinants need not necessarily be linked to mutation: exposure of RIF^R^ *Pseudomonas aeruginosa* mutants to sublethal concentrations of the translational inhibitors, STR and chloramphenicol, has been shown to decrease the fitness cost of a RIF^R^-conferring mutation (as determined by relative growth rate), whereas drugs targeting cellular processes other than translation did not.^86^ As the authors of this study argued, it is possible that the interdependence of transcription and translation ensures that a decreased demand for RNAP activity in the presence of non-lethal translational inhibition reduces the fitness defect of RIF^R^ mutations. If so, this implies that STR^R^ mutations in *rpsL* which reduce ribosome functionality could interact similarly with impaired RpoB activity.^86^ It will be interesting, therefore, to establish whether this relationship is reciprocal; that is, can defective RNAP function mitigate fitness costs that may be associated with STR^R^-conferring mutations?

The propagation of MDR and XDR *Mtb* strains suggests that epistatic interactions might contribute to the phenotypes of strains which, by definition, harbour multiple mutations in essential genes. For MDR-TB, it is generally thought that exposure to both INH and RIF results in the fixation of successive, but distinct, mutations that confer resistance to each drug. However, *in vitro* evidence suggests that the nature of the pre-existing INH^R^ allele can influence the spectrum of subsequent *rpoB* mutations.^87^ Consistent with this idea, analyses of drug resistant clinical *Mtb* isolates have revealed that specific INH^R^ alleles are more frequently associated with resistances to other drugs.^88,89^ While this might reflect the different fitness costs associated with particular INH^R^ mutations,^88^ it is plausible that these data indicate epistatic interactions between INH^R^ and RIF^R^ mutations, a possibility which holds significant implications for the success of MDR-TB strains.

In the most advanced work to date, positive epistasis has been demonstrated between FQ^R^ mutations in *gyrA* and common RIF^R^-associated *rpoB* alleles.^90^ Using the non-pathogenic *M. smegmatis* as surrogate for *Mtb*, mutants harbouring specific *gyrA* and *rpoB* allele combinations were fitter than corresponding strains carrying only a single resistance mutation during competitive growth under standard conditions *in vitro*. Notably, the same *gyrA*/*rpoB* single-nucleotide polymorphism combinations were identified among a panel of clinical XDR *Mtb* isolates, suggesting that epistasis between resistance mutations may be a major factor in the fitness of drug-resistant *Mtb* isolates and, further, might determine the trajectory for the acquisition of multiple resistance mutations.^90^ The established link between transcription rate and DNA supercoiling ^91^ suggests a plausible mechanism for epistatic interactions between mutations in *gyrA* and *rpoB*, though this requires further investigation. More importantly, however, the inferred epistasis signals a need for caution in considering FQs—currently reserved as second-line anti-TB agents—for use in standard regimens for drug-susceptible disease.

## *IN VITRO* FITNESS AS A SURROGATE FOR PATHOGEN SUCCESS?

Drug-resistant *Mtb* strains carrying low fitness cost mutations should, in principle, result in more secondary TB cases. In turn, this has prompted the idea that the prevalence of specific resistance alleles can be equated with fitness: that is, the most common mutations detected clinically are those which incur the smallest fitness cost.^64,73,92,93^ The induction of a fitness hierarchy for specific drug resistance mutations from epidemiological data might not be straightforward, however. In particular, this notion ignores critical uncertainties regarding the nature of the selective forces acting on clinical strains—both during host infection and through the process of strain isolation *in vitro*. For example, while the S531L mutation is frequently observed in clinical *Mtb* isolates, the extent to which other *rpoB* alleles might be subject to strong negative selection *in vitro* is largely unknown, yet could profoundly prejudice apparent strain (and allele) frequencies detected among clinical samples.^94^ Standard media used for strain isolation (and propagation) for routine diagnostic procedures share common constituents with growth media used in experimental assays of competitive fitness; for example, Middlebrook 7H9 base and dextrose are the major components of the major mycobacterial detection platforms^95^ and constitute the preferred culture medium in most mycobacterial research laboratories.^67^ It seems likely, therefore, that a selection bias might be inadvertently imposed through the use of a single, glucose-based growth medium. Critically, the same bias would extend to fitness assays *in vitro*, thereby reinforcing inferred relationships between clinical frequency and capacity for competitive growth.

A recent study applied deep sequencing to examine the diversity of drug resistance mutations in clinical samples.^15^ Instead of sequencing individual colonies, a ‘scrape' of colony forming units was taken from the solid medium on which *Mtb* bacilli had been isolated from sputum. This novel approach uncovered significant levels of heterogeneity of drug resistance-associated mutations within individual patients.^15^ However, it was still dependent on *in vitro* culture of *Mtb* prior to sequencing, which means that only those strains that were culturable on that medium were represented in the analysis. It is possible, therefore, that ‘culture-free' (or metagenomic) techniques will be required to interrogate the potential genetic diversity of the infecting *Mtb* population in sputum and/or other biological samples.

It is also necessary to consider the environmental context when evaluating relative strain fitness. A rare variant of the Beijing lineage, which is differentiated by a small change in its IS*6110* fingerprint, is thought to be less fit owing to the fact that ‘atypical Beijing' strains are infrequently observed in clinical settings. Nevertheless, a RIF^R^ variant sustained transmission in a community with high levels of human immunodeficiency virus (HIV).^96^ Similarly, a RIF^R^ *Mtb* isolate carrying an uncommon *rpoB* mutation was associated with unusually high virulence and transmission in an HIV positive cohort.^97^ It is possible, therefore, that host populations with compromised immunity impose very different pressures on the infecting (and transmitting) organisms, and so might be associated with altered requirements for strain success.^98^

## THE IMPACT OF *RPOB* MUTATIONS ON (MYCO)BACTERIAL PHYSIOLOGY

The potential for drug resistance mutations to alter cellular function seems especially relevant to RpoB as an essential component of the highly conserved bacterial RNAP^99,100^, an idea that is supported by multiple studies demonstrating drug-independent physiological effects of RIF^R^ mutations. In the section below, we briefly review evidence from various bacterial systems that implicates *rpoB* mutations in diverse phenotypic and functional alterations (summarized in Table 1), and discuss their potential relevance to *Mtb*.

### RpoB mutations mimic the stringent response

Antibiotic production in *Streptomyces* sp. (like *Mycobacterium* sp., a genus of the phylum Actinobacteria) is fundamentally dependent on pathways activated by the bacterial alarmones, guanosine tetraphosphate and guanosine pentaphosphate (commonly designated as (p)ppGpp).^104,105,123^ Structural evidence from *Thermus thermophilus* suggests that (p)ppGpp binds near the active site of bacterial RNAPs via an interaction with the β and β′ subunits.^124^ It is interesting, therefore, that *rpoB* mutations corresponding to those found in RIF^R^ *Mtb* isolates are associated with increased antibiotic synthesis by antibiotic-producer strains of *S. coelicolor*.^39,104,105^ Moreover, the same mutations have been shown to induce production of novel antibiotic compounds in *S. coelicolor* strains previously characterized as antibiotic ‘non-producers'.^39^ RIF^R^ mutations have also been shown to activate secondary metabolism, as well as induce (p)ppGpp-independent antibiotic production, in strains that are defective in their inability to produce the alarmone.^105^

The genomes of some *Nonomuraea* and *Nocardia* species contain two closely related but non-identical paralogues of *rpoB*. In these organisms, the second *rpoB* gene confers RIF^R^ and its activation induces the expression of cryptic genes.^117,118,119^ Similarly, heterologous expression of the alternate *Nonomuraea rpoB* gene in *S. lividans* induces antibiotic biosynthesis.^117,118^ Again, it is notable that the sequence polymorphisms that differentiate the second *rpoB* gene from the canonical paralogue correspond to *rpoB* mutations commonly detected in RIF^R^ *Mtb* isolates. In combination, these observations suggest that specific *rpoB* mutations might phenocopy the effects of the (p)ppGpp-mediated stringent response. Consistent with this idea, work in *E. coli* has shown that, in *spoT* and *relA* deletion mutants which are unable to produce (p)ppGpp, RIF^R^ mutations can decrease RNAP stability at promoters that are transcribed during rapid growth, thereby releasing RNAP for transcription at stringently regulated promoters.^38^ This study examined a selection of promoters, so it remains to be established whether other stringent response genes might be regulated in this way. However, these results imply that, while *rpoB* mutations might result in defective growth in nutrient rich environments, the capacity of the mutant alleles to mimic the stringent response may be especially beneficial for competitive survival under nutrient-limited conditions.

The recent observation that (p)ppGpp induces expression of a cryptic STR^R^ determinant in *S. enterica* suggests that, as a result of its broad effects on transcription, this signalling molecule could mediate resistance to other drugs.^125^ Given the functional overlap between certain *rpoB* mutations and (p)ppGpp-mediated transcriptional regulation (discussed above), this raises the additional possibility that RIF^R^ strains might be less susceptible than the wild-type parental strains to other antibiotic classes, as has been proposed elsewhere.^125^ In *Mtb*, abrogation of the stringent response through targeted deletion of *rel_Mtb_* results in impaired survival under starvation conditions *in vitro*^126^ and during chronic infection in a mouse model.^127^ It will be interesting, therefore, to determine whether the starvation phenotype of the *rel_Mtb_* mutant can be complemented by specific *rpoB* mutations.

### Do *rpoB* mutations alter cell wall metabolism?

The physiological implications of different *rpoB* alleles have not been well studied in *Mtb*. There are, however, some recent papers describing the utilization of ‘omics approaches to characterize RIF^R^ strains. By applying liquid chromatography/tandem mass spectrometry to analyse the proteomes of clinical RIF^R^ *Mtb* isolates, Bisson *et al.*^113^ detected differences in the expression profiles of S531L *rpoB* mutants of the *Mtb* Haarlem lineage and H526D mutants of the Beijing lineage compared to corresponding drug-susceptible counterparts with matched spoligotype and restriction fragment length polymorphism patterns. Conserved hypothetical proteins of unknown function were significantly represented among the differentially expresses proteins, which complicates the biological interpretation of these data. Nevertheless, there were striking differences within—and between—Haarlem and Beijing *Mtb* lineages for proteins involved in the biosynthesis and regulation of phthiocerol dimycocerosate, a methyl-branched fatty acid which has previously been implicated in mycobacterial virulence.^128^ In this case, however, the RIF^R^ strains were associated with increased levels of phthiocerol dimycocerosate precursors but not the full-length lipid, an observation that requires further analysis. In a separate study, the fatty acid contents of two *in vitro*-selected RIF^R^ *Mtb* mutants with distinct *rpoB* mutations (S531L and S522L) were analyzed by gas chromatography–mass spectrometry.^114^ Applying this technique, the authors were able to distinguish the respective *rpoB* mutants from one other, as well as their parental strain based on fatty acid composition. Although these studies are united in identifying alterations in fatty acid metabolism as a feature of RIF^R^ strains, the precise implications for *Mtb* pathogenesis are unclear.^113^ Moreover, no whole-genome sequence data were reported for the strains analysed in these studies, so the possibility that other mutations might contribute to the observed phenotypes cannot be excluded.

It seems, therefore, that establishing an unequivocal relationship between *rpoB* genotype and RIF^R^ phenotype will require the site-directed transfer of selected RIF^R^-conferring mutations into sequenced *Mtb* strains. Moreover, if done in the absence of RIF selection, the impact of a specific *rpoB* mutation on lipid metabolism—or any other bacillary function—could be established without the complication of potentially confounding genetic (or even epigenetic) effects. This approach was applied to a clinical isolate of methicillin-resistant *Staphylococcus aureus* that was associated with decreased daptomycin susceptibility following passage in VAN.^121^ Whole-genome resequencing revealed five-point mutations that distinguished the mutant from the parental isolate, including a single mutation in *rpoB*. Sequential transfer of the identified mutations into the parental strain established that the *rpoB* mutation was responsible for the decrease in susceptibility to both VAN and daptomycin and, furthermore, that the mechanism of resistance related to thickening of the cell wall.^121^ The *rpoB* mutation examined in this study was located outside of the RRDR; however, in intriguing follow-up work, it has been reported that the introduction of a RIF^R^-conferring H526Y mutation (located inside the RRDR) into a VAN-sensitive strain also results in increased cell wall thickness and decreased VAN susceptibility.^122,129^

### RIF^R^ mutants and the host–pathogen interaction

The interaction between *Mtb* and its obligate human host is complex and dynamic. As a critical determinant of the clinical outcome of infection, it is understandable that significant resources have been invested in elucidating the mycobacterial^130^ and host immunological ^131,132^ pathways and mechanisms that influence the progression of *Mtb* through the infection cycle. In contrast, the impact of drug resistance on the host–pathogen interaction remains largely unexplored and so represents a key area for future study given the emergence of drug-resistant *Mtb* as a major global health concern.

There are several studies which have examined the response to *Mtb* antigens of peripheral blood mononuclear cells (PBMCs) isolated from MDR-TB patients. In one example, PBMCs from MDR-TB patients were shown to produce lower levels of the pro-inflammatory cytokine, interferon (IFN)-γ, in comparison to healthy controls following exposure to purified protein derivative.^133^ However, since the antigens in purified protein derivative are not specific to *Mtb*,^134^ it is possible that the observed response to purified protein derivative stimulation was complicated by prior vaccination with BCG and/or exposure to other mycobacterial species.^135^ PBMCs from MDR-TB patients have also been associated with decreased production of IFN-γ and tumor-necrosis factor-α following stimulation with the *Mtb*-specific antigen, ESAT6.^136^ Similarly, exposure of PBMCs derived from MDR-TB-infected individuals to a wider set of *Mtb*-specific antigens—specifically, ESAT6, MPT-51 and GlcB—revealed differences in the levels of cytokines produced, as well as the nature of the producer cell populations, whereas CD4^+^ and CD8^+^ T cells from drug-susceptible TB patients produced both IFN-γ and IL-10 in response to all antigens, in cell populations isolated from MDR-TB patients, only CD8^+^ cells produced IFN-γ in response to ESAT6, and IL-10 in response to GlcB and ESAT6.^137^ These results suggest that CD4^+^ T-cell responses are diminished in MDR-TB patients and further, that the outcome of the assay is influenced by the selection of stimulatory antigen.

MDR-TB patients are often infected for longer periods than those with drug-susceptible TB. Therefore, it is possible that immune regulation to dampen the inflammatory response partially explains the decreased immune response observed in MDR-TB patients. Consistent with this idea, several studies of individuals with prolonged MDR-TB disease have revealed increased levels of transforming growth factor β, IL-10 and regulatory T cells, all of which are involved in suppressing the inflammatory response.^138,139^ In turn, this raises a conundrum regarding causation: does an impaired inflammatory response allow the development of drug-resistant *Mtb* strains in specific hosts, or do resistance-associated mutations alter *Mtb* physiology to the extent that the host–pathogen interaction is affected? Resolving this question is complicated by the fact that, by definition, MDR strains harbour more than a single resistance-conferring mutation and, potentially, carry multiple additional single-nucleotide polymorphisms.^10,11^ It seems critical, therefore, to examine the impact of individual drug resistance mutations—including *rpoB* alleles—on immunological function.

### Do *rpoB* mutations alter competitive growth under stress conditions?

The relative frequency of RIF^R^ mutants in slow- or non-growing bacterial populations has been widely used as a quantitative measure of the capacity of some organisms to increase mutation rates under stress.^140,141,142,143^ However, pioneering work by Wrande *et al.*^75^ demonstrated that clonal expansion of certain *rpoB* alleles during competitive growth underlies the (erroneously) inferred generation of RIF^R^ mutants through stress-induced mutagenesis. That is, *rpoB* mutants propagate owing to a selective advantage in stress conditions,^75^ a phenomenon recently extended to nalidixic acid-resistant *gyrA* mutations.^144^ While the mechanisms remain to be determined, the identification of ‘cheater' *rpoB* mutants reinforces the idea that RIF^R^ alleles might be selected in the absence of drug pressure. In turn, this raises the possibility that RIF^R^ mutants of obligate pathogens such as *Mtb* might be associated with different epidemiological prevalence owing to altered disease dynamics, something that requires further investigation.

Further evidence of the potential role of *rpoB* mutations in modulating growth phenotypes is provided by work in *Bacillus subtilis*, which has shown that the spectrum of RIF^R^-conferring mutations differs significantly according to growth state.^101^ Moreover, single amino acid changes in *rpoB* have been associated with major metabolic changes in the same organism, enabling the utilization of diverse substrates that had not previously been identified as suitable for supporting growth.^103^ For example, a S531L mutant was better able to use a variety of β-glucosides, compounds that are likely to be prevalent in the soil owing to breakdown of plant material. As the authors note, this suggests that specific *rpoB* alleles might be beneficial in environments where β-glucosides represent the primary carbon source and, therefore, could drive the competitive expansion of RIF^R^ clones under nutrient-limited conditions.^103^

Multiple studies have implicated *rpoB* mutations in the adaptation of *E. coli* to *in vitro* stress.^110,111,145^ For example, whole-genome resequencing identified *rpoB* and *rpoC* among a handful of mutated genes following extended growth of *E. coli* in glycerol minimal medium.^110^ Critically, transfer of the mutant alleles into a wild-type *E. coli* background confirmed that the *rpoB* and *rpoC* mutations conferred the greatest increase in growth rate in the same medium.^110^ These observations were validated in follow-up work, which showed that mutants containing at least one *rpoB* or *rpoC* polymorphism were associated with better fitness under nutrient limiting conditions, reinforcing the role of RNAP mutations in the adaptation of *E. coli* to environmental stress.^108^ More recently, the same group has demonstrated that *rpoC* mutations are associated with alterations in transcriptional elongation and pausing—and therefore, gene expression—which suggests this as a plausible molecular mechanism for the observed metabolic changes and growth adaptation.^109^ In related work, *E. coli* populations were subjected to different stressors in an investigation of short-term evolution.^145^ Again, this study identified *rpoB* mutations in strains that were better adapted to carbon limitation, n-butanol, osmotic and acid stress.^145^ Similarly, a separate study identified *rpoB* mutants among those strains that had adapted to elevated temperatures.^111^ In combination, these observations establish the contribution of *rpoB* (and *rpoC*) mutations to altered metabolic capacity and further, reinforce the idea that RIF^R^-associated mutations might emerge in the absence of drug selection.

## FUTURE PROSPECTS

**Figure 2:**
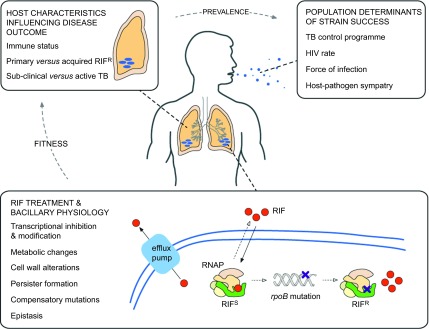
Factors influencing the success of RIF^R^ *Mtb* strains. Although the focus of this review is on RIF resistance in *Mtb*, many of the themes are relevant to other drugs and other infectious organisms. RIF treatment and bacillary physiology: within a single bacterial cell, many factors contribute to the development and maintenance of drug resistance. The drug (in this case, RIF) enters the cell by passive diffusion and, once in the cytoplasm, must translocate and bind to its target (here, RNAP). Some organisms encode enzymes that inactivate RIF,^147^ while recent work suggests that RIF is actively extruded by efflux pumps in *Mtb*.^112,148^ The concentration of RIF that is available to bind to RNAP (that is, the effective intracellular concentration) is a major determinant of whether resistance mutations develop or not, and is heavily influenced by the mechanisms described above;^149^ therefore, the ability to measure this parameter accurately^150^ will be critical to future efforts to understand the development of drug resistance. As described in the main text, mutations in *rpoB* might alter the physiology of the RIF^R^ bacterium. Host characteristics influencing disease outcome: any physiological alteration has the potential to influence the interaction of the bacillus with its obligate human host. Similarly, multiple host factors such as age, nutritional status and copathologies will determine infection outcomes, including transmission to other susceptible individuals. Population determinants of strain success: there is an additional layer of complexity when considering the spread of organisms within and between host populations. Factors such as HIV prevalence, force of infection and socioeconomic status will influence the ability of the organism to transmit between hosts.^151^ While these elements are grouped separately in the figure, it is likely that multiple factors will overlap to influence the success of different *Mtb* strains.

Mutations in *rpoB* have been associated with altered physiology and metabolic function in a variety of bacterial systems. It seems likely, therefore, that equivalent RIF^R^-associated mutations in *rpoB* might be under dual selection in *Mtb*: that is, the combined benefits of RIF^R^ and the physiological advantage(s) of the causal *rpoB* allele might fix *rpoB* mutants in the infecting *Mtb* population (Figure 2). In turn, this raises key questions for future research. For example, do *rpoB* alleles impact disease pathology? Can RIF^R^-associated mutations alter transmissibility and/or the ability of MDR strains to establish an infection? Do mutations in other drug targets (e.g., *gyrA*) influence physiology, as demonstrated recently in *Salmonella*?^85,146^ If so, how do different combinations of resistance mutations influence pathogen success? Addressing these and related questions will be critical in determining the full impact of RIF^R^—and other drug-resistance alleles—on *Mtb*, a pathogen whose persistence in the human population suggests a unique ability to adapt to dynamic and often hostile host environments.

## Figures and Tables

**Table 1:** Impact of RIF^R^-associated *rpoB* mutations on bacterial physiology

Organism	Observed phenotype	Reference
*B. subtilis*	The spectrum of *rpoB* mutations in spores is distinct from those found in vegetative populations and is similar to those associated with clinical *Mtb* isolates	101
	Widespread changes in globally regulated processes such as competency, germination and sporulation	102
	The ability to metabolise substrates previously thought to be non-utilisable for *B. subtilis* and other important changes to *B. subtilis* carbon source metabolism	103
*B. subtilis* and *S. coelicor*	Increased antibiotic production and production of cryptic or previously unobserved secondary metabolites	39,104,105,106
*E. coli*	In strains unable to produce (p)ppGpp, *rpoB* mutations mimic (p)ppGpp regulation and a ‘stringent'-like phenotype is observed.	38,107
	Widespread changes in mechanistic aspects of transcription, including pausing, termination and affinity for nucleotides during elongation	40,42,44,45
	Temperature sensitivity and phage susceptibility	43
	Adaptation to minimal medium, predominantly as a result of a mutation in *rpoB* or *rpoC*	108,109,110
	Growth advantage during stationary phase growth	75
	Significant epistatic interactions between antibiotic resistance-associated mutations in *rpoB*, *rpsL* and *gyrA*	84
	Evolution of RIF^R^ *rpoB* mutations in response to thermal stress in the absence of RIF	111
*Mtb*	Spectrum of *rpoB* mutations changes when the pH of a chemostat culture is lowered	63
	After exposure to RIF, strains containing *rpoB* mutations have increased ofloxacin minimum inhibitory concentrations	112
	Alteration important cell wall components such as phthiocerol dimycocerosates and fatty acid precursors	113,114
	Increased *dnaE2* expression	115
*N. meningitidis*	Decrease in cell membrane permeability	116
*Nonomurea* and *Nocardia*	A second *rpoB* gene confers RIF^R^ and activates expression of dormant genes	117,118,119
*P. aeruginosa*	Differential carbon source metabolism	86
	Fitness of *rpoB* mutants is increased when treated with sub-inhibitory concentrations of protein synthesis inhibitors	86
*S. aureus*	Better biofilm formation on catheters in mice, and a distinct set of mutations observed during murine infection compared to *in vitro* growth	120
	Mutations in *rpoB* are observed in both clinical and lab-derived isolates that have increased vancomycin resistance	121,122
